# Development and Cross‐Sectional Validation of the Quality of Life and Function Five‐Domain Scale (QFS‐5) in Dementia‐Free Community‐Dwelling Older Adults

**DOI:** 10.1002/gps.70215

**Published:** 2026-04-23

**Authors:** Zahinoor Ismail, Ibadat Warring, Dylan X. Guan, Pierre Chue, Andrea Bardell, Clive Ballard, Byron Creese, Anne Corbett, Ellie Pickering, Pamela Roach, Eric E. Smith

**Affiliations:** ^1^ Hotchkiss Brain Institute University of Calgary Calgary Alberta Canada; ^2^ Cumming School of Medicine University of Calgary Calgary Alberta Canada; ^3^ Department of Clinical Neurosciences University of Calgary Calgary Alberta Canada; ^4^ Department of Community Health Sciences University of Calgary Calgary Alberta Canada; ^5^ O'Brien Institute for Public Health University of Calgary Calgary Alberta Canada; ^6^ Department of Psychiatry University of Calgary Calgary Alberta Canada; ^7^ Department of Pathology and Laboratory Medicine University of Calgary Calgary Alberta Canada; ^8^ Clinical and Biomedical Sciences Faculty of Health and Life Sciences University of Exeter Exeter UK; ^9^ Department of Psychiatry University of Alberta Edmonton Alberta Canada; ^10^ Department of Psychiatry University of Ottawa Ottawa Ontario Canada; ^11^ Department of Psychiatry University of British Columbia Vancouver British Columbia Canada; ^12^ Brunel University London UK; ^13^ Department of Family Medicine University of Calgary Calgary Alberta Canada

**Keywords:** EQ‐5D, function, older adults, QFS‐5, quality of life (QoL), validation

## Abstract

**Introduction:**

Quality of life (QoL) captures objective life conditions, subjective wellbeing, and personal aspirations. Interest is growing in ability‐based, patient‐centered instruments, not confounded by health outcomes. The Quality of Life and Function Five Domain Scale (QFS‐5) uses a multi‐dimensional approach to measure QoL emphasizing abilities and life engagement. We describe the development and validation of the QFS‐5 in a large sample of community‐dwelling older adults.

**Methods:**

The validation sample comprised 1610 participants aged ≥ 50 from the Canadian Platform for Research Online to Investigate Health, Quality of Life, Cognition, Behavior, Function, and Caregiving in Aging (CAN‐PROTECT). Internal consistency was assessed using Cronbach's alpha and item‐total correlations. Confirmatory factor analysis (CFA) evaluated domain structure. Criterion validity was tested through Pearson's correlation with the EuroQol‐5D (EQ‐5D). Convergent and discriminant validity were evaluated from associations with cognitive, mental health, and functional measures. Floor and ceiling effects were investigated.

**Results:**

The QFS‐5 demonstrated excellent internal consistency (*α* = 0.92); CFA supported the proposed domain structure with strong item loadings. Criterion validity was confirmed with correlation of −0.61 against the EQ‐5D; higher symptom burden on related scales were associated with lower QFS‐5 scores. Floor effects were minimal, while modest ceiling effects were observed in some domains. No significant floor or ceiling effects were found in participants with frailty.

**Conclusion:**

Validity and reliability are established for this ability‐based QoL scale, within a sample of mostly cognitively unimpaired, community‐dwelling older adults. The QFS‐5 aligns with EQ‐5D, demonstrating potential clinical and research utility to measure relevant patient‐reported QoL outcomes.

## Key Points

- The QFS‐5 is a valid and reliable measure of quality of life, demonstrating excellent internal consistency, strong factor structure, and good alignment with the established EQ‐5D.
- The QFS‐5 captures ability‐based, patient‐centered outcomes, supporting scale utility for research and clinical assessments.

## 1 Introduction

Quality of life (QoL) is defined by the World Health Organization (WHO) as how individuals perceive their overall wellbeing, based on personal goals, values, and cultural context [1]. QoL is a holistic construct that takes objective life conditions, subjective feelings of wellbeing, and personal aspirations into consideration to provide a multi‐dimensional perspective on health‐related burden [2]. As populations age and the prevalence of diseases, such as Alzheimer disease, rise, the accurate measurement of QoL in older adults has become essential in clinical and research settings [3]. For example, by 2050, an estimated 150 million people will be living with dementia—an almost 100 million person increase from 2019 [4]. Dementia is projected to cost the world economy $14.5 trillion, imposing a significant burden on individuals, caregivers, and healthcare systems [5]. In the context of dementia research, QoL offers a meaningful lens to assess wellbeing, serve as a potential marker of disease‐related burden, and provide personal and functional level insights [6].

Traditional QoL instruments often take a deficit‐based approach, with a focus on disease‐related symptoms and/or health states [7], which limits generalizability and obscures insights into function and life engagement. The concept of QoL is frequently used interchangeably with health and functional status, resulting in the mixed use of negative and positive life components including death and function respectively [7, 8]. For example, the EuroQol‐5D (EQ‐5D) measures QoL through the dimensions of mobility, self‐care, usual activities, pain/discomfort, and anxiety/depression [9]. The EQ‐5D has been shown to effectively measure QoL among individuals with dementia, Parkinson disease, cancer, and cardiovascular disease [10, 11, 12, 13, 14].While pain/discomfort can provide insights into patient health status, the construct measures impairment instead of QoL [15]. As described by the disability paradox, a patient with chronic pain may report high QoL due to strong social supports but a pain‐free patient might report low QoL due to isolation [16]. Additionally, depressive symptoms are often included in QoL assessments, but depression is also associated with poor QoL, which results in a conceptual overlap in models that incorporate both mental health symptoms and QoL [17]. To accurately assess the impact of clinical symptoms on patient well‐being, measurement tools should be conceptually distinct from the conditions being evaluated.

In response to the lack of multifaceted QoL measurement tools, there is a growing interest in instruments that capture ability‐based, patient‐centered outcomes (PROs) across life domains without being confounded by specific health outcomes [18]. The Quality of Life and Function Five Domain Scale (QFS‐5) was developed to meet this growing need. The QFS‐5 is a five domain scale generated to measure QoL with a focus on abilities and life engagement [19]. The scale evaluates five key domains—productivity, self‐care, social relationships, leisure and activities, and life satisfaction—reflecting aspects of life that are commonly valued across cultural and demographic backgrounds, irrespective of pain or medical/psychiatric conditions [20, 21]. Rather than a focus on deficits, the QFS‐5 focus is on ability and engagement, with an a priori decision not to query any physical or mental health symptomatology. While the EQ‐5D and QFS‐5 serve distinct methodological purposes, the QFS‐5 enables the assessment of associations between physical or mental health conditions and QoL without conflating health status (i.e., pain, anxiety/depression) with QoL itself. By capturing broader perceptions of life engagement beyond health‐related domains, the QFS‐5 avoids conceptual overlap and measurement confounding that may occur when health is embedded within QoL constructs. The result is broader applicability across chronic and mental health conditions.

We aim to outline the development and validation of the QFS‐5 in a large cohort of community‐dwelling, mostly cognitively unimpaired older adults. Through analyses of reliability, factor structure, criterion and construct validity, and floor and ceiling effects, we provide evidence that the QFS‐5 is a robust and valid instrument for assessing QoL. The QFS‐5 has potential to complement traditional clinical metrics while maintaining research applicability and be used as a measure of QoL in the general population.

## 2 Materials and Methods

### 2.1 QFS‐5 Development

A modified Delphi technique was used, comprising iterative rating rounds and discussion rounds to address points of disagreement. The goal was to create a concise, modern, abilities‐based QoL scale suitable for use as a patient‐reported outcome (PRO) in a waiting room or research settings, irrespective of diagnosis [22]. A pre‐specified domain structure was used comprising productivity, self‐care, leisure and activities, relationships, and life satisfaction and engagement. An a priori decision was made to base the measure on abilities in day‐to‐day life, rather than specific disabilities.

Phase 1 involved generation of QoL domains. The developers (ZI, PC, AB) identified domains based on literature reviews and clinical experience, starting with the well‐established Occupational Therapy (OT) model comprising Productivity, Self‐care, and Leisure. These domains are considered the three primary areas of human occupation, intrinsically linked to QoL because they fulfill essential human needs for meaning, identity, health, and participation in society [23]. OT views engagement in these occupations as fundamental to well‐being. The Social Relationships domain was included due to substantial evidence and a seminal review supporting social relationships as a critical determinant of QoL [24]. The Life Satisfaction/Life Engagement domain was added based on literature directly linking life evaluation (satisfaction) and hedonic well‐being (engagement) to QoL. A combination of population‐level and biological evidence (e.g., links to cardiovascular function, inflammation) suggested that life satisfaction and positive engagement were not just outcomes but contributors to overall quality of life and healthspan [25]. Support for Life Engagement also came from our experience developing Life Engagement PROs as meaningful outcomes in clinical trials [20, 26, 27]. Utilizing these categorical measures in a domain structure offers conceptual simplicity while emphasizing a balance among domains.

Phase 2 involved populating domains with questions. Each developer generated 3‐5 specific, identifiable questions (items) for each of the 5 domains, resulting in an initial pool of ∼40 items. Redundant questions were removed or combined, leaving 30 items. These items were then reviewed and refined collectively to optimize clarity and relevance. Phase 3 involved voting and item reduction using a 3‐point alignment scale (strong, moderate, weak). Alignment was based on relevance, uniqueness, utility, and familiarity/experience of the clinician with the item. Weakly aligned items were removed, and moderately aligned items were either modified or removed. Consensus was achieved over 3 rounds of iterative refinement, informed by pragmatic compromise, i.e., the final scale format incorporated elements from each panelist's preference, fostering collective ownership of the final 5‐domain 25‐item. As mental and physical health items (e.g., depression, pain) are strongly linked to QoL [28], these were purposefully excluded in order to avoid potential confounds in persons/populations with mental or physical health problems. The justification for this decision was that by excluding syndrome‐ or disease‐specific items (e.g., depression, pain), the QFS‐5 could be used agnostic to syndrome or disease in multiple populations and settings, without direct confounds in samples with mental or physical health symptoms.

### 2.2 Questionnaire Description

The QFS‐5 comprises 25 items distributed equally across five domains: (1) Productivity (e.g., I can maintain a job, family responsibilities); (2) Self Care (e.g., I live independently without supports); (3) Social Relationships (e.g., I feel like I am part of a community); (4) Life Satisfaction (e.g., I am able to enjoy myself); and (5) Leisure and Activities (e.g., I participate in active hobbies like playing music, games, reading). A 4‐point frequency‐based metric (never, sometimes, frequently, always) measures how often one was able to satisfy the question in each item over the last month. Items within each domain are summed to acquire a domain total score (0–15), and all domain total scores are summed to acquire a QFS‐5 total score (0–75), with higher scores indicating higher QoL.

### 2.3 Validation (Psychometric Testing) Study Design

We used baseline data from the Canadian Platform for Research Online to Investigate Health, Quality of Life, Cognition, Behavior, Function, and Caregiving in Aging (CAN‐PROTECT, www.can‐protect.ca) study for the cross‐sectional validation of the QFS‐5 [29]. CAN‐PROTECT is a nation‐wide digital epidemiology platform for dementia‐free participants ≥ 18 years of age, and study partners complete yearly assessments on demographics, health, cognition, behavior, function, lifestyle, QoL, and other factors related to health and aging. A convenience sample of participants across Canada was recruited through local communication channels, social media platforms, community events, and dementia advocacy organizations, with ongoing digital data collection. Recruitment started in March 2023 up to April 2024 for this analysis. Recruitment is ongoing as are annual assessments for longitudinal analyses. CAN‐PROTECT has proven effective for gathering data on risk and resilience in brain aging [30, 31, 32, 33, 34, 35, 36, 37, 38]. CAN‐PROTECT received ethics approval from the Conjoint Health Research Ethics Board at the University of Calgary (REB21‐1065). The study protocol has been published [30].

### 2.4 Participants

**FIGURE 1:**
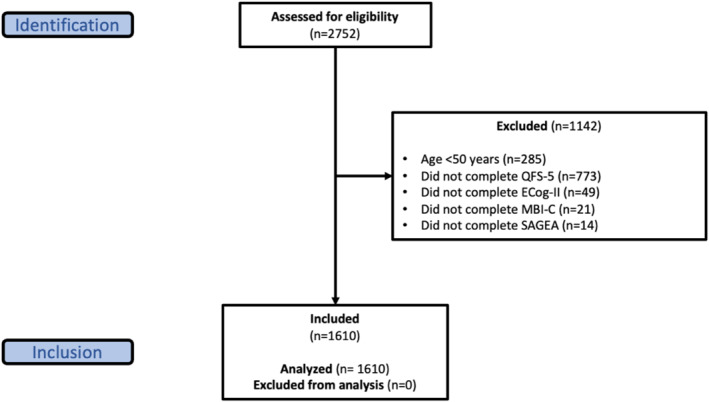
Participant flow diagram. Quality of Life and Function Five Domain Scalet; Ecog‐II, Everyday Cognition Scale; MBI‐C, Mild Behavioral Impairment Checklist; SAGEA, Standard Assessment of Global Everyday Activities.

For the validation, 2752 participants with baseline data were assessed for eligibility. The present validation study included 1610 participants aged ≥ 50 years who completed the QFS‐5 in addition to the relevant assessments on demographics, cognition, behavior, and function. Although CAN‐PROTECT enrolls adults ≥ 18 years, the present validation focused on participants aged ≥ 50 years to align with a CAN‐PROTECT aim of studying aging‐related changes in cognition, function, and QoL. A flow diagram detailing the inclusion/exclusion criteria is shown in Figure 1.

### 2.5 Statistical Analysis

R version 4.3.2 was used to conduct all statistical analyses. Participant characteristics were summarized using means, standard deviation (SD), and ranges for numerical variables and counts with percentages for categorical variables. Demographics were collected including participant age (years), sex (male/female), education (years), ethnocultural origin, and marital status (yes/no). Ethnocultural origin was not mutually exclusive with multi‐select options consisting of European, North American, Caribbean, South American, African, Asian, and Oceanic origins.

Item‐level analysis was conducted to assess item contributions to the overall scale using Cronbach's alpha, which was derived to measure internal consistency. A higher alpha coefficient is indicative of greater reliability, which suggests that the items measure the intended QoL construct. Item‐total correlation measured the correlation between each item and total score. The adjusted *r* indicates the correlation between each item and the QFS‐5 total score calculated after removing that item from the total. The adjusted correlation was used to identify potentially irrelevant items and assess whether scale reliability could be improved by removing the items from the scale. Higher correlations indicated that the item was related to the construct of QoL being measured.

Test‐retest reliability and inter‐rater reliability could not be assessed. CAN‐PROTECT data are collected yearly, too long an interval to effectively evaluate test‐retest reliability. Additionally, the QFS‐5 is only self‐reported in CAN‐PROTECT so inter‐rater reliability could not be assessed.

To validate the domain structure of the QFS‐5 defined through development, we conducted a confirmatory factor analysis and assessed fit using the Root Mean Square Error of Approximation (RMSEA) and Comparative Fit Index (CFI). Existing guidelines indicate that excellent fit thresholds are RMSEA ≤ 0.06 and CFI ≥ 0.95 [39].

Criterion validity was evaluated through comparison with the EQ‐5D, a widely used instrument known for its simplicity and brief administration [40]. The EQ‐5D measures QoL, where higher scores indicate poorer QoL [40]. To assess criterion validity, a Pearson's correlation was performed. Pearson's correlation measures the strength of a linear relationship between the EQ‐5D and QFS‐5. To establish construct validity, we evaluated convergent and discriminant validity for the QFS‐5. Convergent validity was determined by examining the linear relationship between the QFS‐5 total score and other measures known to be strongly associated with QoL, including the Everyday Cognition‐II (ECog‐II) scale [41, 42], Mild Behavioral Impairment Checklist (MBI‐C) [31, 43], and the Standard Assessment of Global Everyday Activities (SAGEA) [37]. These instruments measure changes in cognition, behavior, and function, respectively, often due to disease or age‐related changes, which are relevant to overall QoL [42, 44, 45]. All scale scores were obtained by summing all items across an entire scale (total) or scale domain. Discriminant validity was determined by examining the relationship between the QFS‐5 total score and sex, a construct not thought to significantly influence QoL. Convergent and discriminant validity were both modeled using linear regressions with the QFS‐5 total score as the outcome variable and no included covariates.

To assess content validity and data completeness, floor and ceiling effects were analyzed. According to established guidelines, a floor or ceiling effect is present if more than 15% of participants achieve the lowest or highest possible score, respectively [46, 47]. To determine whether these effects were present in a sample of older adults with health conditions, we assessed floor and ceiling effects in a subsample of individuals with frailty (*n* = 166). Frailty was operationalized based on the Fried phenotypic criteria, which include unintentional weight loss, weakness, poor endurance and energy, slowness, and low physical activity levels [48]. Participants were classified as frail if they met three or more of these criteria.

## 3 Results

### 3.1 Descriptive Statistics

The validation included 1610 participants (mean age: 64. 7 ± 7.6 years, 80.2% female). Participants were highly educated with a mean of 15.8 ± 4.5 years of education [Table 1]. In the frail subsample (*n* = 166), the mean age was 65.9 ± 8.3 years with 84.3% being female. Means, SDs, and item‐total correlations (unadjusted and adjusted) for each item are shown in Table 2. In general, item scores tended to be right skewed with means > 2.0 and SDs ranging from 0.33 to 1.01. All items showed satisfactory or near‐satisfactory adjusted item‐total correlations (i.e., correlation between the score of each item and the total score of all other items excluding the item itself) of > 0.31 [39], suggesting that no items needed to be deleted or significantly modified.

**TABLE 1:** Participant characteristics.

Variable	Total
*n*	1610
Age (years)	64.7 (7.6), 50–94
Sex (female)	1292 (80.2)
Education (years)	15.8 (4.5), 0–29.5
Ethnocultural origins	
European	1364 (84.7)
North American	761 (47.3)
Caribbean	12 (0.7)
South American	11 (0.7)
African	15 (0.9)
Asian	53 (3.3)
Oceanic	8 (0.5)
Marital status (married)	1249 (77.6)
QFS‐5 score	
Total	65.5 (9.2), 19–75
Productivity	14.2 (1.4), 3–15
Self‐care	14.1 (1.6), 3–15
Social relationships	12.5 (2.8), 0–15
Life satisfaction	13 (2.6), 1–15
Leisure and activities	11.7 (3), 2–15
EQ‐5D total score	6.9 (2), 5–19
ECog‐II total score	12.3 (12), 0–99
MBI‐C total score	5.5 (7.5), 0–65
SAGEA score	3.1 (3.9), 0–27

*Note:* Participant characteristics were summarized using means, standard deviation, and ranges for numerical variables and counts with percentages for categorical variables. All scale scores were obtained by summing all items across an entire scale (total) or scale domain. Ethnocultural origins are not mutually exclusive.

Abbreviations: EQ‐5D, EuroQol‐5D; Ecog‐II, Everyday Cognition Scale; MBI‐C, Mild Behavioral Impairment Checklist; QFS‐5, Quality of Life and Function Five Domain Scale; SAGEA, Standard Assessment of Global Everyday Activities.

**TABLE 2:** QFS‐5 items and descriptive statistics.

Item	Mean	SD	*r* (unadj)	*r* (adj)
Productivity				
I am able to maintain a job, my family responsibilities, go to school, or volunteer	2.81	0.57	0.50	0.45
I can accomplish the tasks I need to	2.72	0.52	0.60	0.57
I attend and am on time for appointments	2.89	0.35	0.34	0.31
I can take public transportation or drive to where I need to go	2.90	0.41	0.33	0.29
I pay my bills on time	2.90	0.35	0.35	0.31
Self‐Care				
I live independently without supports	2.92	0.39	0.32	0.28
I keep my residence neat and clean	2.62	0.62	0.58	0.53
I keep myself clean and tidy	2.86	0.38	0.54	0.51
I can prepare food and I eat enough	2.92	0.33	0.46	0.43
I can take care of my physical health	2.80	0.50	0.60	0.56
Social Relationships				
I get along with people	2.73	0.48	0.57	0.54
I am able to have an intimate relationship	2.35	1.01	0.54	0.45
I have close friends or relationships	2.59	0.71	0.0.63	0.58
I feel comfortable with people	2.49	0.69	0.68	0.64
I feel like I am part of a community	2.33	0.85	0.74	0.69
Life Satisfaction				
I am able to enjoy myself	2.49	0.67	0.77	0.74
I feel I have a good quality of life	2.63	0.65	0.77	0.74
I have a sense of purpose in life	2.41	0.78	0.73	0.68
I am able to make my own life choices	2.84	0.44	0.54	0.51
I am well supported in my life	2.67	0.62	0.70	0.65
Leisure and Activities				
I participate in active hobbies (e.g., playing music, dance, crafts, games, reading)	2.45	0.76	0.66	0.61
I participate in passive hobbies (e.g., listening to music, watching television or using my computer/mobile device/gaming console)	2.73	0.50	0.38	0.32
I can participate in learning activities for things I enjoy	2.33	0.81	0.66	0.60
I engage in sports, clubs, or leisure activities	1.96	0.97	0.67	0.61
I am able to engage in relaxation activities or exercises	2.19	0.86	0.68	0.62

*Note:* Unadjusted item‐total correlation coefficient (r) indicates correlation between each item and the QFS‐5 total score calculated without dropping the item score from the total score. The adjusted r indicates the correlation between each item and the QFS‐5 total score calculated after dropping the item.

Abbreviations: QFS‐5, Quality of Life and Function Five Domain Scale.

### 3.2 Reliability

The QFS‐5 achieved a Cronbach's alpha = 0.92 (95% CI: 0.91−0.92) suggesting excellent internal consistency. Dropping any single item from the QFS‐5 did not noticeably change the reliability of the scale (all alpha = 0.91 or = 0.92) suggesting that the removal of any one item would not significantly improve reliability.

### 3.3 Factor Analysis

Confirmatory factor analysis results indicate an excellent fit with an RMSEA of 0.04 and a near‐satisfactory fit with a CFI of 0.94. These findings support the proposed domain structure of the QFS‐5. Factor loadings indicated strong relationships between each item and their corresponding domain (all item loadings > 0.30) [Table 3]. All domains were significantly correlated with each other (all *p* < 0.001).

**TABLE 3:** Factor loading of each QFS‐5 item onto its domain.

Item	Loading	SE	*p*
Productivity			
I am able to maintain a job, my family responsibilities, go to school, or volunteer	1.00		
I can accomplish the tasks I need to	1.13	0.08	< 0.001
I attend and am on time for appointments	0.43	0.6	< 0.001
I can take public transportation or drive to where I need to go	0.42	0.06	< 0.001
I pay my bills on time	0.42	0.07	< 0.001
Self‐Care			
I live independently without supports	1.00		
I keep my residence neat and clean	2.85	0.48	< 0.001
I keep myself clean and tidy	1.78	0.300	< 0.001
I can prepare food and I eat enough	1.34	0.22	< 0.001
I can take care of my physical health	2.42	0.37	< 0.001
Social Relationships			
I get along with people	1.00		
I am able to have an intimate relationship	1.65	0.13	< 0.001
I have close friends or relationships	1.69	0.11	< 0.001
I feel comfortable with people	1.87	0.10	< 0.001
I feel like I am part of a community	2.48	0.15	< 0.001
Life Satisfaction			
I am able to enjoy myself	1.00		
I feel I have a good quality of life	0.99	0.03	< 0.001
I have a sense of purpose in life	1.10	0.03	< 0.001
I am able to make my own life choices	0.45	0.03	< 0.001
I am well supported in my life	0.82	0.06	< 0.001
Leisure and Activities			
I participate in active hobbies (e.g., playing music, dance, crafts, games, reading)	1.00		
I participate in passive hobbies (e.g., listening to music, watching television or using my computer/mobile device/gaming console)	0.35	0.03	< 0.001
I can participate in learning activities for things I enjoy	1.04	0.04	< 0.001
I engage in sports, clubs, or leisure activities	1.31	0.05	< 0.001
I am able to engage in relaxation activities or exercises	1.17	0.05	< 0.001

*Note:* The factor loading of the first item of each domain was fixed at 1 as they were used to set the scale of each factor.

Abbreviations: QFS‐5, Quality of Life and Function Five Domain Scale.

### 3.4 Validity

**FIGURE 2:**
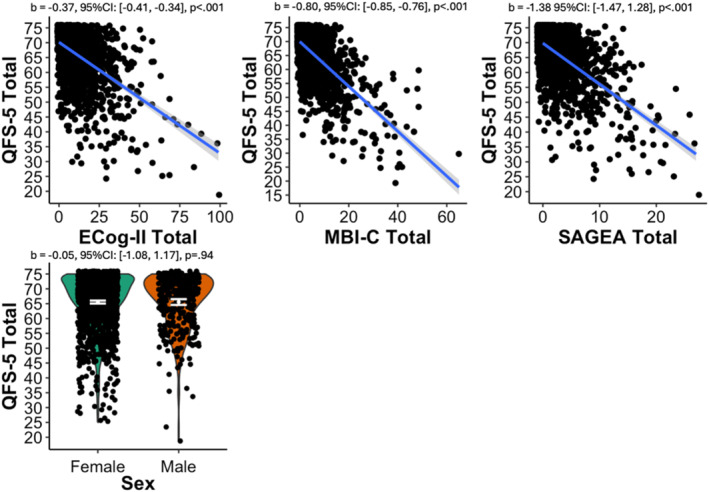
Convergent and discriminant validity of the QFS‐5. Quality of Life and Function Five Domain Scale; Ecog‐II, Everyday Cognition Scale; MBI‐C, Mild Behavioral Impairment Checklist; SAGEA, Standard Assessment of Global Everyday Activities.

A correlation coefficient of *r* = −0.61 (95% confidence interval (CI): −0.64 to −0.58) was derived to assess criterion validity of the QFS‐5 against the EQ‐5D. We also found that higher scores on the ECog‐II, MBI‐C, and SAGEA were significantly associated with lower QFS‐5 scores (ECog‐II: *b* = −0.37, 95% CI: −0.41 to −0.34, *p* < 0.001; MBI‐C: *b* = −0.80, 95% CI:−0.85 to −0.76, *p* < 0.001; SAGEA: *b* = −1.38, 95% CI:−1.47 to −1.28, *p* < 0.001) [Figure 2]. The analysis further showed that sex was not significantly associated with QFS‐5 scores [Figure 2].

Floor and ceiling effects can be found in Table 4. For the total QFS‐5 score, no notable floor effects (0.06%) were observed, and ceiling effects remained below the threshold (13.04%). In specific domains, no floor effects were observed. However, there were notable ceiling effects in domain‐specific scores. In the subsample of frail individuals, no notable floor (0.60%) or ceiling (1.20%) effects were observed in the total QFS‐5 score [Table 4]. Furthermore, no floor effects were found in any individual domain. In the frail subsample, ceiling effects were observed in the productivity, self‐care and life satisfaction domains. No ceiling effects were observed in the social relationships or leisure and activities domains.

**TABLE 4:** Floor and ceiling effects in QFS‐5 total score and domains.

Domain	Total sample (*n* = 1610)	Total sample	Frail subsample (*n* = 166)	Frail subsample
	Floor effect (%)	Ceiling effect (%)	Floor effect (%)	Ceiling effect (%)
Total QFS‐5 score	0.06	13.04	0.60	1.20
Productivity	0.06	64.22	0.60	34.34
Self‐care	0.06	60.62	0.60	27.71
Social relationships	0.06	34.16	0.60	12.05
Life satisfaction	0.12	43.35	0.12	15.06
Leisure and activities	0.37	25/15	1.81	4.82

*Note:* All scale scores were obtained by summing all items across an entire scale (total) or scale domain.

Abbreviations: QFS‐5, Quality of Life and Function Five Domain Scale.

## 4 Discussion

In a sample of mostly cognitively unimpaired community dwelling functionally independent older persons, the QFS‐5 is successful in measuring QoL through establishing validity, reliability, and effective use of domain structure. Criterion, convergent, and divergent validity were verified through comparison with established measures including the EQ‐5D. These findings highlight the potential of the QFS‐5 as a practical self‐report measure that can complement measurement‐based care approaches in older persons. To further establish reliability, future studies should consider evaluating test‐retest reliability and inter‐rater reliability.

Measurement‐based care is increasingly recognized as a valuable framework to monitor progress, guide clinical decisions, and optimize outcomes [49]. Systematic and routine use of rating scales has been shown to improve treatment planning and patient engagement [50]. However, in clinical practice, rating scale use remain low due to factors including patient concerns about confidentiality, practitioner beliefs prioritizing clinical judgment, lack of resources for training, and competing requirements [31, 51]. Self‐report measures like the QFS‐5 offer an opportunity to involve patients directly in their care, promote efficiency, and reduce clinician burden. Moreover, QoL is often more reliably self‐reported than symptoms, especially in populations where insight into illness may be limited [52, 53].

Criterion validity assessed whether the operationalization of the QFS‐5 predicts the construct of QoL. The strong negative correlation coefficient (*r* = −0.61) indicates that higher scores on the QFS‐5, which reflect better QoL, are associated with lower scores on the EQ‐5D, which also reflect better QoL, thereby supporting criterion validity of the QFS‐5. The significant correlation establishes that the QFS‐5 effectively measures the intended construct in comparison to the already well‐established EQ‐5D. Furthermore, the magnitude of the observed association suggests that the QFS‐5 may serve as a feasible alternative while maintaining comparability. The comparability of the two scales is further demonstrated in a study showing similar associations between mild behavioral impairment, a dementia risk marker, and QoL when assessed with the EQ‐5D and the novel QFS‐5 [31].

While the EQ‐5D remains a widely accepted reference measure, an important limitation is the inclusion of the anxiety/depression dimension [54, 55]. The incorporation of a mental health component may confound the measurement of overall QoL [28, 56], particularly in populations where mood disturbances are prevalent or fluctuating [57]. This dimension can introduce bias or conflate correlations between QoL and psychiatric symptoms, making it difficult to disentangle the impact of physical functioning and general well‐being from emotional states. For example, individuals experiencing depressive symptoms may report disproportionately lower scores that do not necessarily reflect their broader QoL. Further, one cannot assess the independent impact of depression on QoL if the QoL measure includes depression. We made the explicit decision to exclude symptom‐based items, allowing the QFS‐5 to measure perceived ability and engagement across domains, across multiple conditions, and avoiding overt confounds.

The confirmatory factor analysis provides robust evidence supporting the structural validity of the QFS‐5. The model fit, indicated by the RMSEA and CFI, demonstrate that the proposed domain structure aligns with the observed data. Notably, all factor loadings exceeded the 0.30 threshold, indicating that each item meaningfully contributes to the intended domain. The significant intercorrelations among domains further reinforce that while the domains are conceptually distinct, they represent interconnected aspects of QoL, consistent with the multidimensional framework proposed in scale development.

Convergent validity was used to establish the QFS‐5 among other measures that assess similar constructs including the ECog‐II, MBI‐C, and SAGEA. The negative correlations suggest that as participants experience difficulties in relation to cognition, behavior, and function, their perceived QoL as measured by the QFS‐5 decreases [Figure 2]. The association suggests that the QFS‐5 effectively measures constructs related to QoL. In contrast, divergent validity tests whether the QFS‐5 is correlated with unrelated constructs such as sex. The lack of association between the QFS‐5 and sex [Figure 2] supports the discriminant validity of the QFS‐5, demonstrating that it measures constructs related to QoL without being confounded by unrelated/demographic factors.

The absence of floor effects across all domains suggests that very few participants scored at the lowest possible level, indicating that the QFS‐5 can effectively measure a range of low to moderate functioning. While no significant floor effects were observed, the ceiling effects suggest that a substantial proportion of participants scored at the highest possible level in each domain, indicative of higher domain‐related QoL scores. This observation aligns with the study sample being a non‐clinical population comprised mostly of community dwelling functionally independent older adults with no major complaints about QoL. To further explore floor and ceiling effects, a subsample of frail individuals was utilized. Despite the presence of ceiling effects in three domain‐specific QFS‐5 scores, there is an overall reduction in ceiling effects compared to the overall sample. The reduction in ceiling effects in the QFS‐5 among individuals with frailty indicate the ability of the scale to effectively measure QoL across various medical conditions and clinical syndromes. These findings are promising, as they suggest that the QFS‐5 may be sensitive among individuals with higher levels of health vulnerability. Future studies targeting more clinically complex populations (e.g., cancer, multiple sclerosis, epilepsy) may help establish whether ceiling effects persist or are mitigated in contexts of greater impairment.

The QFS‐5 was intentionally developed as an ability‐based measure, enabling application across the aging continuum, including cognitively unimpaired individuals, those at risk for cognitive decline or frailty, and clinical populations such as Alzheimer disease. Measuring QoL in community‐dwelling older adults is particularly important for identifying early unmet needs, such as reduced engagement, declining independence, or social isolation, which may precede cognitive or functional impairment. Additionally, a strength of the QFS‐5 is that psychiatric and behavioral symptoms do not confound the modeling.

Other abilities‐based measures have been developed, including Life Satisfaction Index (LSI‐A), published in 1961, which set a precedent for measuring positive affect, outlook, and life evaluation [58].The 20‐item scale also includes zest, congruence between desired and achieved goals, and mood tone, thus straying from the goals of the QFS‐5. World Health Organization Quality‐of‐Life Scale (WHOQOL‐BREF), a 26‐item scale addressing the domains of social relationships, and physical, psychological, and environmental health over a 2‐week window [59]. However, affective symptoms are still queried [60]. The Quality‐of‐Life Inventory (QOLI) is a proprietary scale that provides a weighted QoL score based on satisfaction and importance across 16 domains (e.g., health, money, creativity, children) over the last month. In addition to high cost, other barriers include psychometric, scoring, and statistical concerns stemming from the weighted multiplicative score [61].

Given demographic limitations, future work should assess the scale in diverse patient populations to ensure generalizability. The study sample was predominately female and comprised mostly of highly educated participants with at least some North American or European ancestry, which was based on multi‐select ethnocultural origins [Table 1]. Given that participants had high educational attainment, they may not be fully representative of the broader Canadian population and might present with systematically different health‐seeking behaviors and/or access to care compared to underrepresented groups. Future research should prioritize targeted recruitment strategies, including partnerships with primary care clinics, community health centers, and culturally specific community organizations, to ensure sample diversity and generalizability of findings. Future studies should also incorporate both sex and gender. Given that clinical and research assessments increasingly move to digital platforms, it is critical that scales can be reliably completed online, ensuring accessibility across diverse populations. Future studies should assess longitudinal sensitivity to change, further evaluate scale performance in clinical populations with higher symptom burden (both medical and psychiatric), and build evidence to demonstrate the scale's reliability.

## 5 Conclusion

The development and validation of the QFS‐5 address important limitations in existing QoL scales, which may be confounded by symptom burden, and lack of specificity to patient perceptions. In contrast, PROs like the QFS‐5 can engage patients more directly in their care, are efficient to administer, and less influenced by clinician biases or those of care partners/informal caregivers, who often rate QoL lower than patients themselves [62]. Given the importance of assessing outcomes that matter to patients, such as QoL and function, the QFS‐5 holds promise as an accessible, patient‐centered tool for routine clinical use and research applications. Future studies and validations will clarify the research and clinical populations best served by the QSF‐5 and the utility of the domains in identifying potential targets for intervention.

## Ethics Statement

Ethics approval for the study was obtained from the Conjoint Health Research Ethics Board at the University of Calgary (REB21‐1065).

## Conflicts of Interest

ZI has served as advisor/consultant for Eisai, Lilly, Lundbeck/Otsuka, Novo Nordisk, and Roche. AB has received consulting and speaking fees from Abbvie, Otsuka, Lundbeck, and Janssen. CB has no direct disclosures related to the current paper. With respect to broader disclosures. CB has received consultancy honoraria from Acadia, BMS, TauRx, Maplight, Abbvie, Lilly, Janssen, NovoNordisk, Biogen and Roche pharmaceutical companies over the last 3 years.

## Data Availability

The data that support the findings of this study are available on request from the corresponding author. The data are not publicly available due to privacy or ethical restrictions.
